# Carcinoembryonic Antigen Serum Levels in Nonmelanoma Skin Cancer

**DOI:** 10.3390/biomedicines6010024

**Published:** 2018-02-23

**Authors:** Saverio Latteri, Vito Emanuele Catania, Giulia Malaguarnera, Andrea Peri, Gaetano Bertino, Giuseppe Frazzetto, Antonio Maria Borzì, Antonio Biondi, Rosario Emanuele Perrotta, Michele Malaguarnera

**Affiliations:** 1 Department of Medical, Surgical Sciences and Advanced Technologies “G.F. Ingrassia”, University of Catania, 95123 Catania, Italy; savlat@tiscali.it; 2 Department of Biomedical and Biotechnological Science, University of Catania, 95123 Catania, Italy; giulia.malaguarnera@live.it (G.M.); michele.malaguarnera@gmail.com (M.M.); 3 Research Centre “The Great Senescence”, University of Catania, 95120 Catania, Italy; giuseppefrazzetto@gmail.com (G.F.); antoniomaria.borzi@gmail.com (A.M.B.); 4 Department of General Surgery, Policlinico “San Matteo”, University of Pavia, 27100 Pavia, Italy; a.peri@smatteo.pv.it; 5 Hepatology Unit, Department of Clinical and Experimental Medicine, University of Catania, 95123 Catania, Italy; gaetanobertinounict@gmail.com; 6 Department of General Surgery and Medical-Surgery Specialties, University of Catania, 95100 Catania, Italy; abiondi@unict.it (A.B.); r.perrotta@unict.it (R.E.P.)

**Keywords:** carcinoembryonic antigen, non-melanoma skin cancer, synchronous tumour, metachronous tumour, multiple tumours

## Abstract

Background: Carcinoembryonic antigen (CEA) is a glycoprotein, which is present in the foetal colon, some benign conditions and different malignancies, particularly in colon adenocarcinoma. We focused this study on non-melanoma skin cancer (NMSC). NMSC is a common malignancy and it is an important source of morbidity and death in the world. In this study we evaluated whether CEA level increases in NMSC. Patients and Methods: A total of 566 patients with non-melanoma skin cancer (NMSC) were enrolled; 286 patients with NMSC showed CEA levels above normal values, and 280 showed CEA levels below normal values. Patients with high levels of CEA underwent abdominal ultrasound, gastro endoscopy, colonoscopy, and abdominal CT scans. Results: We studied 566 patients, 286 were positive to CEA and 280 were negative. Of the 286 patients positive to CEA, 132 had basal cell carcinoma (64 patients had an associated cancer) and 154 had squamous cell carcinoma (75 patients were affected by cancer). Of the 280 patients negative to CEA, 130 had basal cell carcinoma (12 were associated with cancer), and 150 had squamous cell carcinoma (18 were associated with cancer). The mean age of the 566 case control subjects were 65–81 years. Of the 10 subjects that were the positive control for CEA, two had cancer. Of the 556 subjects that were the negative control for CEA, three had cancer. Conclusions: In patients that present high serum levels of CEA, we give attention to adenocarcinoma tumour first. The pattern of association may be attributable to bias because the group with NMSC were frequently evaluated than those with no history of NMSC. Our results showed that out of 286 patients that were CEA-positive, 139 had cancer, and of the 280 that were CEA-negative, 30 had cancer. Therefore, 20% of patients do not follow the trend. Other markers should be investigated.

## 1. Introduction

Non-melanoma skin cancer (NMSC) is one of the most common forms of human malignancy [1,2]. The formation of this cancer may be related to environmental carcinogens [3], genes, and immunity.

Approximately 80% of NMSC are basal cell carcinomas and 20% are squamous cell carcinomas [4,5]. This NMSC is not life threatening, but it is a major source of morbidity and causes more than 1000 deaths annually in the United States [6,7]. Despite the low mortality, because so many patients are diagnosed with NMSC each year, it still has a significant impact on quality of life and is placing a large financial burden on health care services [8].

Basal and squamous cell carcinomas (BCC and SCC) are the two most common skin cancers. Squamous cell carcinoma (SCC) has a higher potential for local recurrence and regional or distant metastasis and individuals with SCCs have a higher overall mortality rate than those with basal cell carcinoma (BCC) [9]. BCC has local invasion and rarely metastasizes [10,11]. Patients with BCCs or SCCs commonly ask what their changes are for developing another one. The risk of developing a second SCC within three years of having one is about 18%; the risk of developing a second BCC within three years of having a BCC (or SCC) is about 44%. The risk of developing a SCC, in patients with prior BCC, is low (6% within three years).

Two potential pathways that may relate NMSC risk to overall cancer risk are DNA repair and inflammatory and/or immune response pathways, which have been hypothesized to be linked to carcinogenesis in general, making it plausible to speculate that impaired immunity results in an increased risk for multiple cancers, including NMSC. In the gastroenterological setting, NMSC might have an increased risk with inflammatory bowel disease and gastrointestinal tumours [12,13,14,15,16,17].

NMSC is easily curable with surgical excision and is rarely fatal [18]. Despite such a favourable prognosis, there is evidence that NMSC may be a marker of other adverse health outcomes. An association between NMSC and increased risk of other malignancies has been well documented [19,20].

The tumour marker was applied to a number of clinical questions related to diagnosis, staging, monitoring, and prognosis of carcinoma. Serum tumour markers, such as carcinoembryonic antigen (CEA) and/or carbohydrate antigen, are used for cancer detection in clinical practice. CEA is one of the most widely-used tumour markers worldwide and, certainly, the most frequently-used marker in colorectal cancer and other carcinomas.

Carcinoembryonic antigen (CEA) is a complex, highly-glycosylated macromolecule. The glycoprotein that belongs to the immunoglobulin superfamily is identified in both foetal colon and colon adenocarcinoma, but it appears to be absent from the healthy adult colon. Since the protein was detected only in cancer and embryonic tissue, it was given the name carcinoembryonic antigen (CEA). CEA is present in columnar epithelial cells and goblet cells of the tongue, oesophagus, and cervix, and in secretory epithelia and duct cells of sweat glands [21,22].

The human CEA gene family is clustered on chromosome 19q and comprises 29 genes. Of these 18, seven are expressed belonging to the CEA subgroup and 11 to the pregnancy-specific glycoprotein subgroup. The gene encoding is a member of the immunoglobulin supergene family. CEA expression in the above-mentioned organs commence during the early foetal period and seem to persist throughout life.

The CEA or CEA-like molecule is also present in certain healthy tissues, although the concentration in tumours was, on average, 60-fold higher than in the non-malignant tissues. In the skin, CEA has been accepted as a marker for sweat gland differentiation [23]. Since CEA is a stable molecule, has a fairly restricted expression in normal adult tissue and is expressed at high levels in positive tumours. The bulk of CEA in a healthy individual is produced in the colon where it is released from the apical surface of mature columnar cells into the gut lumen and disappears with the faeces [24,25].

Although in vitro data implicate CEA in cell adhesion, its localization to the apical surface of mature enterocytes in healthy human colon is difficult to reconcile with this role. In the healthy colon, CEA has been found to bind certain strains of *Escherichia coli*. According to Thompson (1991), this binding may facilitate bacterial colonization of the intestine [26]. Hammarström suggested that CEA might play a role in protecting the colon from microbial infection, possibly by binding and trapping infectious microorganisms [27].

Tumour markers are widely used in oncology and have become an integral part of the clinical management of many different malignancies. Due to the ease of measuring serum biomarkers, the relatively low cost makes CEA the most widely-used biomarker. CEA has roles in screening, diagnosis, as well as monitoring the results of treatment [28]. The objective of this study is to evaluate if CEA has a predictive value in gastro-intestinal tumours associated with NMSC.

## 2. Methods and Materials

### 2.1. Patients

We performed a case control study. Cases included 566 patients in total with NMSC and 466 controls. The patients’ characteristics are described in Table 1. Patients and control subjects underwent to abdominal ultrasound and, if needed, computed tomography. Both patients and matched subjects performed clinical analysis and CEA. Ineligible patients were those with prior histories of another cancer, severe jaundice, pulmonary renal chronic diseases, liver disease, prostatic diseases, autoimmune diseases, diabetes mellitus, pancreatic cancer, and gastrointestinal cancer. All sensitive data were collected and protected with respect of present privacy statements. The patients enrolled in this study were admitted to Cannizzaro Hospital during the period from 2010 to 2016.

Eligible patients for this prospective longitudinal study were those who were 60 years of age or older, with the presence of non-melanoma skin cancer. A total of 566 patients with non-melanoma skin cancer, confirmed histologically, underwent examination, which included measurement of CEA. A total of 286 patients with non-melanoma skin cancer showed CEA above normal values, and 280 showed CEA below normal values. For each case of non-melanoma skin cancer one CEA-positive control was randomly chosen from risk sets consisting of all cohort members alive and non-melanoma free. Matching criteria were country, sex, date of blood collection (± one year relaxed to ± five years) and those subjects with dermatitis admitted to our hospital for colonoscopy or gastroscopy. Clinical evaluations, hematochemical, virological, instrumental, and histological analysis were performed on these patients [28].

### 2.2. Ethics

Study recruitment was performed in observation and respect of the Helsinki Declaration. All patients and control subjects gave their informed written consent for the study participation and for each invasive procedure they underwent.

### 2.3. Methods

NMSC was subdivided into basal cell and squamous cell carcinomas. The reference population is situated in relatively homogeneous areas regarding sun exposure. The average number of sunny hours per years is between 1200 to 1800 h.

Before the diagnostic procedures were undertaken approximately 10 mL of venous blood was drawn from each patient and left for 20 min at room temperature. Then the samples were centrifuged at 1000 rpm for 10 min.

### 2.4. Laboratory Analysis

The following serum analyses were performed: azotaemia, creatinine, AST and ALT (aspartate aminotransferase and alanine aminotransferase), γGT (gamma glutamil-tranferase), alkaline phosphatase, prothrombin, fibrinogen, and prothrombin time (PT).

### 2.5. Tumour Marker Assay

Blood samples (10 mL each) were taken from patients, processed, and stored at −80 °C in our serum bank until examination. Serum assay for CEA was performed with an Immulite 2000 assay (Diagnostic Product Corporation, Los Angeles, CA, USA). Immunolite 2000 CEA is a solid-phase, two-site sequential chemiluminescent immunometric assay. To prevent erroneous results due to the presence of fibrin or lipemic samples, we ensured that complete clot formation had taken place prior to centrifugation and, if recommended, to ultracentrifuge the samples. To assess the effect of sample types blood was collected from 10 volunteers and was spiked with various concentration of CEA to obtain values throughout the calibration range of the assay. The cut-off value in malignant and benign tumours was <5 ng/mL. The tumour report includes information of clinical morphological or laboratory examination. All cases were individually matched by gender, age, and calendar year of diagnosis with subjects.

## 3. Statistical Analysis

Continuous variables have been presented with mean values ± standard deviation (SD). Numerical variables were expressed as median and range. The Kolmogorov-Smirnov test was used to assess the normal distribution of CEA serum levels. A comparison between serum levels was obtained by means of the Mann-Whitney *U* test. Differences in frequencies for categorical variables were assessed using the Fisher exact test.

We analysed the data obtained and calculated sensitivity, specificity, positive predictive value (PPV), negative predictive value (NPV), positive likelihood ratio (PLR), and negative likelihood ratio (NLR).

Receiver operating characteristic (ROC) curve analysis: the ROC curve was obtained by calculating the sensitivity and specificity of a test at every possible cut-off point and plotting sensitivity against 1-specificity. The curve was used to select the optimal cut-off value for a test result.

The diagnostic accuracy of the test was measured by the test was measured by the area under the curve (AUC), which were tested using AUC = 50% as a reference.

## 4. Results

### 4.1. Basal Characteristic of NMSC

**Figure 1:**
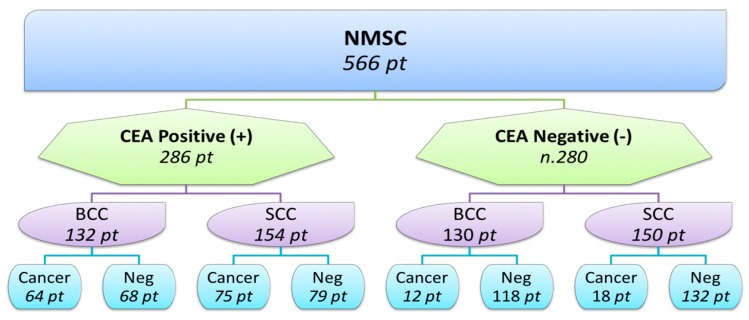
Representation of CEA patients’ distribution. pt: patients.

NMSC was located on the face 346, chest in 88 patients, a leg in 57 patients, the scalps in 49 patients, the hands in 11 patients, the buttocks in eight patients, and the neck in seven patients. Of these patients 286 were positive for CEA and 280 were negative. Regarding the histology, 304 patients were affected by squamous cell carcinoma, and 262 by basal cell carcinoma. Of the 286 patients positive for CEA, 132 had basal cell carcinoma and 154 had squamous cell carcinoma. Of the 280 patients negative for CEA, 130 had basal cell carcinoma and 150 had squamous cell carcinoma (Figure 1).

Of the 10 control subjects, positive for CEA, two had cancer. Of the 556 control subjects, negative for CEA, three had cancer.

#### 4.1.1. CEA in the Squamous Cell Carcinoma

CEA serum levels in the squamous cell carcinoma were positive in 154 patients: 75 patients were affected by cancer. CEA was negative in 150 patients: 18 were associated with cancer (Figure 1). The CEA sensitivity is 80% (C.I. 75–84%); specificity, 62% (C.I. 56–68%); and PPV, 48% (C.I. 43–54%), (Table 2).

#### 4.1.2. CEA in the Basal Cell Carcinoma

CEA serum levels in the basal cell carcinoma were positive in 132 subjects: 64 CEA-positive patients had an associated cancer; 130 subjects were CEA-negative: 12 had associated cancers (Figure 1). The CEA sensitivity is 84% (C.I. 79–88%); specificity, 63% (C.I. 57–69%); and NPV, 88% (C.I. 83–91%).

#### 4.1.3. CEA in the Matched Control

In the matched control CEA serum levels were positive in 10 subjects: two were affected by tumours. CEA serum levels were negative in 556 subjects: three were affected by tumours (Figure 1).

The CEA sensitivity is 40% (C.I. 33–47%); specificity, 98% (C.I. 96–99%); PPV, 20% (C.I. 16–24%); and NPV, 99% (C.I. 97–99%).

##### Comparison with Groups

In the comparison with NMSC and dermatitis group we observed a higher value in CEA (*p* < 0.001) and in SBP (*p* < 0.001), in BPB (*p* < 0.01), in weight (*p* = 0.014), in AST (*p* < 0.001), in ALT (*p* < 0.001), in total bilirubin (*p* < 0.001), in fasting glucose (*p* < 0.01), in serum urea, and a significantly lower value in gamma-GT (*p* < 0.001), in ALP (*p* < 0.001), and in creatinine.

##### Comparison with SCC and Dermatitis

In the comparison between SCC to dermatitis groups we observed higher values in CEA (*p* < 0.001) and in SBP (*p* < 0.001), in AST (*p* < 0.001), in ALT (*p* < 0.001), in FG (*p* < 0.001), and lower values in weight (*p* < 0.001), gamma GT (*p* < 0.001), and ALP (*p* < 0.001).

##### Comparison between BCC to Dermatitis

In the comparison between BCC to dermatitis we observed higher CEA serum levels in the BCC group (*p* < 0.001), in BMI (*p* < 0.02), in SBP (*p* < 0.001), in weight (*p* < 0.01), in AST (*p* < 0.001), in ALT (*p* < 0.001), in FG (*p* < 0.001), and in urea (*p* < 0.001), and lower serum levels (*p* < 0.001) in gamma GT, ALP, and serum creatinine.

##### Comparison between SCC to BCC

In SCC compared to BCC we observed higher CEA serum levels in SBP (*p* < 0.001), in DBP (*p* < 0.01) in FG (*p* < 0.006), in weight (*p* < 0.01), in urea, and in serum creatinine (*p* < 0.001), and lower in weight (*p* < 0.001).

## 5. Discussion

The association between NMSC and other malignancies was observed among those in both BSC and SCC groups. Measurement of serum tumour markers is an often-used method for cancer risk management. However, each tumour marker test has been developed for a specific category of anticipated cancer. Screening tests reveal no serologic sign of malignancy; however, some tests reveal suspicious results requiring further cancer surveying, with some patients receiving a cancer diagnosis. Screening of cancers has received attention in developed and developing countries, owing to the heavy economic and quality of life burden caused by cancer.

The CEA serum levels are in NMSC *p* < 0.001. Normal serum concentrations of CEA are considered to be lower than 5 ng/mL. It is not uncommon to find that CEA levels can be elevated in diagnosing cancer in patients with gastric, pancreatic, breast, and male genitourinary cancer.

The risk factors for skin cancers include both environmental and genetic factors. NMSC is associated with cumulative sun exposure risk, rather than the intensity of the exposure, as is seen with melanoma. The therapeutic options, such as cryotherapy, ingenol mebutate, imiquimod cream, electrochemotherapy and, recently photodynamic therapy, have improved the prognosis of these pathologies [29]. The photodynamic therapy can be used not only to achieve complete eradication of the NMSC while presenting aesthetically and functionally-important structures, but can also be used in elderly patients and in immune-depressed subjects [30,31,32,33]. Additionally, it is important to treat actinic keratosis that represents the earliest manifestation of NMSC [34,35].

Several studies have found that patients with non-melanoma skin cancer have an increased risk for other types of cancer. Non-melanoma skin cancer (NMSC) was also associated with non-Hodgkin lymphoma, cancer of the breast, colon cancer, prostate cancer, and lung cancer. Several studies have reported that individuals diagnosed with NMSC have higher subsequent or prior diagnoses of secondary primary malignancies, by about 20–60% [36,37].

CEA may be elevated in a wide variety of conditions and the diagnostic accuracy is diminished in these circumstances. Clinical interpretation of CEA measurement requires an appreciation of the normal biology of this tumour marker. CEA is also produced in the goblet cells and released together with the mucins, thus also being present in the outer mucinous layer directly on top of the apical glycocalyx.

For the colon, in which organ the microbial load is the highest, the following arguments for a role in innate immunity can be made: the molecules are located at a most strategic position in the apical glycocalyx facing the microbial environment in the gut.

CEA plays a role in immune defence, protecting the colon, and perhaps other areas, like the upper alimentary tract, the urinary bladder, and the skin (sweat glands), from microbial attack. Despite early promise, CEA has failed to gain an established role in clinical practice, partly due to uncertainty of the predictive value of a positive test, with 48% in BCC and in SCC, and 20% in dermatitis. It is now appreciated that this marker is not exclusively associated with malignant processes, and elevated circulating levels have been reported in a wide range of benign conditions, including liver disease, cholangitis, and pancreatitis.

It is not uncommon to find that CEA levels can be elevated in patients with non-malignant liver disease, because the liver is the main site for CEA metabolism. Moreover, smoking has been found to affect the serum concentration of CEA [38]. With the exception of smoking and hepatic disorders, the specificity of CEA could be greatly improved as a cancer screening tool. Since CEA is a stable molecule, has a fairly restricted expression in normal adult tissue, is expressed in normal adult tissue, and is expressed at high levels in positive tumours, serum CEA tests have little value for screening purposes since the number of false positive tests is too high.

In the association between BCC and gastrointestinal cancer, the high CEA serum levels positively shows a 90% negative predictive value (48% positive value, 84% sensitivity, and 63% specificity). It is reasonable that any noxa (viral or toxic) which is able to promote tissue inflammatory damage and, sequentially, reparative features with fibrotic tissue deposition and parenchymal regeneration. The results of the population-based study suggest that there may be increased baseline risks of BCC in certain individuals with IBD, such as men with Chron Disease.

## 6. Conclusions

We would condition the use of a single measurement of this marker in the routine work-up of patients. The role of planned serial measurement will need to be established by ongoing work, although it is possible to draw some tentative conclusions from the findings of this study [39,40,41,42].

If diagnostic uncertainty persists, one possibility may be to relieve the jaundice suggestive of an underlying malignancy, although a falling value does not exclude the diagnosis. In addition, Ca 19-9 can be used to screen the disease process in patients with gastrointestinal tract cancers who had no elevation of CEA levels. Ca 19-9 can be used to diagnose pancreatic, gastric, and colorectal cancer [43,44,45,46]. Another study has been conducted on four immunochemistry markers simultaneously, including epithelial membrane antigen (EMA), cluster of differentiation 10 (CD10), B-cell lymphoma 2 (Bcl-2), and CEA, to distinguish between BCC and SCC. All of these markers are helpful in routine microscopy [41]. Elevated CEA levels are often observed in smokers, in cancer patients with a variety of non-malignant diseases, and inflammatory conditions.

This study has several limitations. The main limitation of this study is that the subjects are drawn from a tertiary care hospital and, thus, may not fully reflect the general population of non-melanoma skin cancer. For instance, they may have been referred to a specialized centre because they were more symptomatic or evident. If individuals with a history of NMSC were more likely than those without such history to receive regular, high-quality medical care that enhanced the likelihood of cancer detection, this difference would represent a potential source of bias that could contribute to the observed associations.

The pattern of associations that we observed if attributable to surveillance bias and not to a true increase in cancer risk could result if the group with confirmed NMSC had greater access to health care and were evaluated for cancer more frequently than those with no personal history of NMSC.

Overall our results showed that, out of 286 patients were CEA positive, 139 had cancer, and of 280 patients that were CEA negative, 30 had cancer. Therefore, other markers should be investigated in association with CEA in order to reduce the false negatives (20% of patients who did not follow the trend).

## Figures and Tables

**Table 1:** Baseline characteristics of the study cohort (values are expressed as the mean ± SD. The body-mass index (BMI) is the weight in kilograms divided by the square of the height in meters) and the *p*-value. --: values were not evaluated; bold means significant value; N.S.: not significant values.

Characteristics	Dermatitis (n. 566)	Group 1 SCC (n. 304)	Group 2 BCC (n. 262)	Controls vs. Group 1	Controls vs. Group 2	Group 1 vs. Group 2	Controls vs. Group 1 + 2
Sex (Male/Female)	210/256 (45.06%/54.94%)	148/156 (48.68%/51.32%)	113/149 (43.13%/56.87%)	**--**	**--**	**--**	**--**
Age (years)	65–81	65–81	65–81	**--**	**--**	**--**	**--**
Race	Caucasian	Caucasian	Caucasian	**--**	**--**	**--**	**--**
Body-mass index (kg/m^2^)	24.10 ± 2.40	24.20 ± 2.10	24.50 ± 2.20	N.S.	**0.027**	**0.098**	N.S.
Systolic blood pressure (mmHg)	134.20 ± 8.10	138.00 ± 7.80	137.00 ± 8.10	**<0.001**	**<0.001**	N.S.	**<0.001**
Diastolic blood pressure (mmHg)	85.60 ± 9.00	88.00 ± 9.50	86.00 ± 8.90	**<0.001**	N.S.	**0.010**	**0.010**
Weight (kg)	69.10 ± 2.40	68.00 ± 2.30	71.20 ± 2.80	**<0.001**	**<0.001**	**<0.001**	**0.014**
Aspartate Transaminase (AST) (IU/l) (n.v. 8–18)	18.20 ± 2.40	24.10 ± 2.20	23.10 ± 2.00	**<0.001**	**<0.001**	**<0.001**	**<0.001**
Alanine Transaminase (ALT) (IU/l) (n.v. 8–18)	16.60 ± 2.10	21.90 ± 2.40	21.80 ± 2.60	**<0.001**	**<0.001**	N.S.	**<0.001**
Gamma-glutamyltransferase (γGT) (IU/l) (n.v. 2–30)	24.90 ± 4.80	22.70 ± 3.40	22.80 ± 3.20	**<0.001**	**<0.001**	N.S.	**<0.001**
Alkaline Phosphatase (IU/l) (n.v. 35–100)	36.80 ± 2.40	35.10 ± 2.40	35.40 ± 2.90	**<0.001**	**<0.001**	N.S.	**<0.001**
Total bilirubin (mg/dL) (n.v. 0.2–1.2)	0.96 ± 0.20	1.04 ± 0.40	1.07 ± 0.30	N.S.	**<0.001**	0.320	**<0.001**
Fasting glucose (mg/dL) (n.v. 74–106)	81.40 ± 7.80	87.80 ± 8.10	83.20 ± 9.60	**<0.001**	**<0.001**	**0.006**	**<0.001**
Serum urea (mg/dL) (n.v. 6–40)	31.40 ± 3.10	36.00 ± 3.10	34.00 ± 3.30	**<0.001**	**<0.001**	**<0.001**	**<0.001**
Serum creatinine (mg/dL) (n.v. 0.7–1.3)	0.80 ± 0.40	0.80 ± 0.30	0.60 ± 0.30	N.S.	**<0.001**	**<0.001**	**<0.001**
CEA (ng/mL) (n.v. <5)	4.20 ± 1.20	96.10 ± 30.10	68.00 ± 30.30	**<0.001**	**<0.001**	**<0.001**	**<0.001**

**Table 2:** Analysis of CEA serum positivity (n.v. < 5). --: values were not evaluated.

	BCC		SCC		Dermatitis	
	%	CI 95% (LL-UL)	%	CI 95% (LL-UL)	%	CI 95% (LL-UL)
Sensitivity (SE)	84.2	(79.1–88.3)	80.6	(75.7–84.8)	40	(33-47.2)
Specificity (SP)	63.4	(57.3–69.2)	62.6	(56.8–68)	98.3	(96.5-99.2)
Positive Predective Value (PPV)	48.5	(42.3–54.7)	48.7	(43–54.5)	20	(16.5–24)
Negative Predective Value (NPV)	90.8	(86.4–93.9)	88	(83.7-91.3)	99.3	(97.9–99.8)
Positive Likelihood Ratio (LR+)	2.303	(1.990–2.666)	2.154	(1.865–2.488)	23.050	(4.399–13.666)
Negative Likelihood Ratio (LR−)	0.249	(0.215–0.288)	0.309	(0.268–0.357)	0.611	(0.209–1.787)
Prevalence	29	(23.7–35)	30.6	(25.5–36.2	1.1	(0.4–2.6)
Odds pre-test −	0.409	--	0.441	--	0.011	--
Odds post-test +	0.941	--	0.949	--	0.250	--
Odds post-test −	0.102	--	0.136	--	0.007	--
P pre-test	0.290	(0.190–0.396)	0.306	(0.214–0.403)	0.011	(−0.076–0.105)
P post-test +	0.485	(0.397–0.573)	0.487	(0.406–0.568)	0.200	(0.121–0.284)
P post-test -	0.092	(−0.018–0.214)	0.120	(0.019–0.231)	0.007	(−0.080–0.101)
